# ID 218 - Monitoramento do Uso de LABA/LAMA para o Tratamento de Pacientes com DPOC no SUS

**DOI:** 10.5327/2237-9622.2025.v34s1.218

**Published:** 2025-11-25

**Authors:** Amanda Oliveira Lyrio, Laís Lessa Neiva Pantuzza, Amanda Oliveira Lyrio, Ana Carolina de Freitas Lopes, Luciene Fontes Schluckebier Bonan

**Keywords:** DPOC, impacto orçamentário, monitoramento pós-incorporação

## Abstract

**Introdução:**

A doença pulmonar obstrutiva crônica (DPOC) é uma condição respiratória crônica que causa obstrução do fluxo aéreo e é progressiva, resultando em dificuldade para respirar. No Sistema Único de Saúde (SUS), o tratamento para os casos graves e muito graves (estágios 3 e 4), com alto risco (grupos C e D), inclui o uso de brometo de tiotrópio monoidratado (LAMA) + cloridrato de olodaterol (LABA) e brometo de umeclidínio (LAMA) + trifenatato de vilanterol (LABA), ambos incorporados em dezembro de 2020. Este estudo tem como objetivo comparar o impacto orçamentário estimado no momento da avaliação de incorporação e o impacto observado desses medicamentos no SUS.

**Método:**

Estudo descritivo com dados de mundo real, retrospectivos, administrativos e nacionais de dispensação, abrangendo o período de 2022 a 2023. Os dados de utilização foram extraídos por meio da Sala Aberta de Situação de Inteligência em Saúde (Sabeis), que é originada do Sistema de Informações Ambulatoriais do SUS (SIA/SUS). Foram incluídos todos os pacientes com registro de uso de brometo de tiotrópio monoidratado (LAMA) + cloridrato de olodaterol (LABA) e brometo de umeclidínio (LAMA) + trifenatato de vilanterol (LABA) para o tratamento de DPOC grave e muito grave. Os preços unitários dos medicamentos foram obtidos no Sistema Integrado de Administração de Serviços Gerais (Siasg) e no Banco de Preços em Saúde (BPS), considerando as compras feitas pelas Secretarias Estaduais de Saúde (SES). O preço médio foi ajustado de acordo com a quantidade adquirida.

**Resultados:**

Os medicamentos umeclidínio + vilanterol e tiotrópio + olodaterol foram incorporados ao SUS em dezembro de 2020, com disponibilização efetiva em janeiro de 2022, 14 meses após a incorporação. O umeclidínio + vilanterol, inicialmente proposto a R$ 70, foi adquirido pelas SES por R$ 87 no primeiro ano e por R$ 83 no segundo, com ressarcimento de R$ 210 pelo governo federal. O tiotrópio + olodaterol, estimado a R$ 197, foi adquirido por R$ 186,14 no primeiro ano e R$ 227,54 no segundo, com ressarcimento de R$ 242. As aquisições foram feitas por revendedores, não diretamente pela empresa. No primeiro ano, a estimativa populacional foi precisa, mas no segundo ano a população observada foi o dobro da estimada. O impacto orçamentário foi 82% menor que o previsto no primeiro ano, mas no segundo ano houve aumento de 68% para tiotrópio + olodaterol e 56% para umeclidínio + vilanterol, refletindo variações no custo e na demanda.

**Conclusão:**

A análise do monitoramento pós-incorporação de LABA/LAMA para tratamento de DPOC no SUS aponta que, apesar da estimativa populacional precisa no primeiro ano, o impacto orçamentário foi muito inferior ao previsto, possivelmente devido à entrada gradual dos pacientes e à suposição de adesão plena desde o início. No segundo ano, tanto a população quanto o impacto foram superiores ao estimado, refletindo uma difusão maior do que era esperado. O preço de aquisição também foi superior ao proposto, provavelmente por conta da compra via revendedores, e não diretamente da empresa. Além disso, o tempo de implementação superou os 180 dias recomendados, ocorrendo 14 meses após a incorporação, o que pode ter influenciado o descompasso entre estimativas e resultados.

